# Quantification of β-lactamase producing bacteria in German surface waters with subsequent MALDI-TOF MS-based identification and β-lactamase activity assay

**DOI:** 10.1016/j.heliyon.2024.e27384

**Published:** 2024-03-05

**Authors:** Lara Stelmaszyk, Claudia Stange, Michael Hügler, Jatinder P.S. Sidhu, Harald Horn, Andreas Tiehm

**Affiliations:** a TZW: DVGW Technologiezentrum Wasser, Department of Water Microbiology, Karlsruher Straße 84, Karlsruhe, Germany; b CSIRO Oceans and Atmosphere, Ecosciences Precinct, 41 Boggo Road, Brisbane, Australia; c Karlsruher Institut für Technologie, Engler-Bunte Institute, Wasserchemie und Wassertechnologie, Engler-Bunte-Ring 9a, Karlsruhe, Germany

**Keywords:** Antibiotic resistance, Environmental bacteria, Culture-based methods, MALDI-TOF MS, qPCR, β-lactamases, Pathogens, Oligotrophic bacteria

## Abstract

Environmental oligotrophic bacteria are suspected to be highly relevant carriers of antimicrobial resistance (AMR). However, there is a lack of validated methods for monitoring in the aquatic environment. Since extended-spectrum β-lactamases (ESBLs) play a particularly important role in the clinical sector, a culturing method based on R2A-medium spiked with different combinations of β-lactams was applied to quantify β-lactamase-producing environmental bacteria from surface waters. In German surface water samples (n = 28), oligotrophic bacteria ranging from 4.0 × 10^3^ to 1.7 × 10^4^ CFU per 100 mL were detected on the nutrient-poor medium spiked with 3^rd^ generation cephalosporins and carbapenems. These numbers were 3 log_10_ higher compared to ESBL-producing *Enterobacteriales* of clinical relevance from the same water samples. A MALDI-TOF MS identification of the isolates demonstrated, that the method leads to the isolation of environmentally relevant strains with *Pseudomonas*, *Flavobacterium,* and *Janthinobacterium* being predominant β-lactam resistant genera. Subsequent micro-dilution antibiotic susceptibility tests (Micronaut-S test) confirmed the expression of β-lactamases. The qPCR analysis of surface waters DNA extracts showed the presence of β-lactamase genes (*bla*_TEM_, *bla*_CMY-2_, *bla*_OXA-48_, *bla*_VIM-2_, *bla*_SHV_, and *bla*_NDM-1_) at concentrations of 3.7 (±1.2) to 1.0 (±1.9) log_10_ gene copies per 100 mL. Overall, the results demonstrate a widespread distribution of cephalosporinase and carbapenemase enzymes in oligotrophic environmental bacteria that have to be considered as a reservoir of ARGs and contribute to the spread of antibiotic resistance.

## Highlights

• β-lactam resistant oligotrophic bacteria are widespread in German surface waters.
• ESBL-*Enterobacteriales* were detected in 3 log-level lower concentration.
• *Pseudomonas*, *Flavobacterium* and *Janthinobacterium* were identified with MALDI-TOF MS.
• Expression of β-lactamases was proofed in resistant environmental isolates.
• Cephalosporine and carbapenem resistance genes were detected in water samples.

## Graphical abstract

**Figure:**
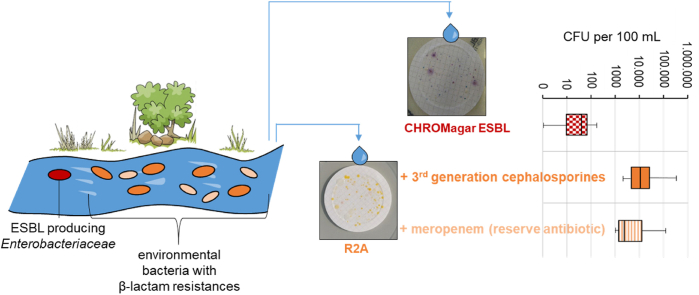

## 1 Introduction

Antibiotics are used in human and veterinarian medicines, but also in animal husbandries including factory farming for food production and aquaculture. Therefore, antibiotics, antibiotic resistant bacteria (ARB), and antibiotic resistance genes (ARGs) are introduced into the environment through the discharge of fermentation-based antibiotic production residues, as well as municipal and clinical wastewater treatment plant effluents [[1], [2], [3]]. Entering environmental habitats, antibiotics can pose a selective pressure on bacteria, even when present in minimal or sub-inhibitory concentrations [4] and thus, lead to accelerated development of intrinsic antibiotic resistances [[5], [6], [7]]. As a consequence, ARGs can be horizontally transferred to other bacteria, leading to better fitness in strongly anthropogenic-influenced environments [[8], [9], [10], [11], [12], [13], [14]]. Therefore, the environment and environmental bacteria have been rated important reservoirs for AMR [15,16].

Identification and implementation of measures to detect AMR transmission pathways is a key element for the management of the global AMR crisis [17,18]. Methods commonly used to detect ARGs in the clinical area and in the environment include PCR-based methods and metagenomic analysis of DNA extracts from microbial communities representing different water matrices [19,20]. With these methodologies, a wide range of ARGs have already been detected in surface waters [[21], [22], [23], [24], [25], [26]]. In addition, culture-based methodologies are used to detect clinically-relevant ARB in surface waters and hospital wastewaters [2,27,28].

Antimicrobial susceptibility tests are generally used to detect resistance patterns and multi-resistances in bacterial isolates. For clinical isolates, disk diffusion and broth dilution methods are used in most laboratories for phenotypic antimicrobial susceptibility testing [29]. An advantage of the broth dilution test is, that numerous antibiotics and drug combinations (e.g. β-lactam-antibiotics in combination with β-lactamase-inhibitors) can be tested on a single panel. Corresponding tests for the clinical settings are already commercially available [30]. However, these broth microdilution tests are rarely used for testing environmental isolates.

In recent years, matrix-assisted laser desorption ionization-time of flight mass spectrometry (MALDI-TOF MS) has emerged as a rapid and cost-effective tool for microbial identification. This technology determines a characteristic proteomic fingerprint of a bacterial strain. By comparison with a reference spectra database, the examined bacterial isolate can be assigned to a genus and species [31]. MALDI-TOF MS has already been applied to identify coliform bacteria in environmental water samples [32]. However, there is limited experience with environmental ARB.

In 2017, the World Health Organization (WHO) published a global priority list of ARB, posing a great threat to human health [33]. In this list, *Acinetobacter baumanii*, *Pseudomonas aeruginosa,* and selected species of *Enterobacteriales* with carbapenem resistances and resistances to 3^rd^ generation cephalosporins are listed as organisms with the highest ("critical") priority [34]. Carbapenems and cephalosporins belong to the group of β-lactam antibiotics and have a β-lactam ring in their molecular structure [35]. β-lactam antibiotics can directly bind to a cell wall building enzyme, responsible for peptidoglycan formation and therefore, inhibit the cell wall synthesis of gram-positive and gram-negative bacteria [36]. With the expression of β-lactamases, bacteria can hydrolyze this β-lactam ring and thus, inactivate the antibiotic substances [36]. There are different types of β-lactam hydrolyzing enzymes but most of them have already evolved to have an extended spectrum of activity (extended-spectrum β-lactamases, ESBLs). In particular, carbapenemase-producing strains have high clinical relevance, as carbapenems are categorized as reserve antibiotics in Germany and many other countries [37].

Previous investigations on the distribution of ESBL-producing bacteria in the environment have primarily focused on clinically relevant bacteria and also *Escherichia coli* are seen as useful indicator organisms to detect AMR in the environment [38]. For such investigations (see Table S1, supplementary material), nutrient-rich media containing more than 20 mg/L (up to 36 mg/L) of organic carbon substrates supplied as protein, yeast, or meat extracts and incubation temperatures between 35 and 37 °C have been used [27,[39], [40], [41], [42], [43], [44], [45], [46], [47], [48]]. However, oligotrophic bacteria from the environment will be rarely isolated under such growth conditions.

To better understand the development and transfer pathways of AMR, all potential reservoirs for ARGs, including environmental bacteria, need to be considered. Investigations with culture-based approaches are particularly important, as they reveal viable ESBL-producing bacteria numbers and functional resistances. The aims of this study were (i) to establish and validate a culture-based method for the detection of β-lactam resistant environmental bacteria, (ii) to apply the new method to German surface waters, (iii) to identify relevant bacterial genera, (iv) to test *in vitro* β-lactamases expression, and (v) to compare the occurrence of ESBL-producing *Enterobacteriales* and environmental ARBs and corresponding ARGs in water samples.

## 2 Materials and methods

### 2.1 Sampling of surface water

Sampling of 500–1000 mL river water was carried out using sterile plastic bottles, which were rinsed with the sample and then filled and stored at 4 °C until filtration (max. 24 h). Six surface water samples from Danube river near Langenau, Baden Wuerttemberg, Germany, four samples from Rhine river near Karlsruhe, Baden-Württemberg, Germany and 9 samples from Ruhr river and Stever river near Muelheim a. d. Ruhr, North Rhine-Westphalia, Germany were investigated in 2019, 2020 and 2021 (as listed also in Table S2, supplementary material).

### 2.2 Screening of β-lactam resistant environmental bacteria

To evaluate the occurrence of β-lactam resistant environmental bacteria, a standardized R2A medium (Merck), firstly described by Reasoner and Geldreich (1985) [49], was spiked with (a) 2 mg/L cefotaxim, 2 mg/L ceftazidim, 4 mg/L cefpodoxim (hereafter abbreviated as BL1) and (b) 2 mg/L meropeneme, 4 mg/L ceftazidim (hereafter abbreviated as BL2). The supplements were chosen according to the WHO priority pathogen list for R&D of new antibiotics [33,50]. The concentrations have been chosen higher compared to recommendations for instance by HPA (UK Health Protection Agency, revised in Ref. [51]) to achieve a more pronounced selection, even if some ARB might not be selectable since they have a lower minimal inhibitory concentration (MIC). However, the chosen concentrations allow a good comparability to other studies [[52], [53], [54], [55], [56], [57]]. Additionally, the R2A medium was used without antibiotics to grow oligotrophic bacteria. Different volumes from 0.05 to 0.5 as well as 1, 10 or 100 mL, depending on the sample type, were vacuum filtered through a filter (0.45 μm pore size, EZ-Pak, Merck Millipore) to obtain a minimum of 1 and a maximum of 200 colonies. The filter was incubated on the agar for 48 h at 21 °C. Longer incubation times (72 h) at temperatures of 12 °C, as well as a 50 % reduced nutrient concentration in the medium were tested in the progress of method establishment and were found to reveal no significant differences. Colony forming units (CFU) per 100 mL were calculated according to DIN EN ISO 8199 by counting the total numbers of colonies on a minimum of two and a maximum of three agar plates of different filtering volumes divided by the total filtering volume. One or more colonies, which were phenotypically distinguishable, were transferred to antibiotic-free R2A agar and again incubated at 21 °C for 48 h for following MALDI-TOF MS (Bruker) based strain identification and further microdilution tests for determining the β-lactamase type expressed by the isolates. Colonies were cryoconserved at −75 °C using the CRYOBANK system (MAST diagnostica).

### 2.3 Screening of antibiotic resistant pathogens and indicator bacteria

To evaluate the occurrence of ESBL-producing *Enterobacteriales*, rated as critical priority pathogens according to WHO (2017) [33], a standardized chromogenic selective medium (CHROMagar™ ESBL, MAST diagnostica) was used, as described previously in Schreiber et al. (2021) [27]. Volumes of 1, 10 or 100 mL were vacuum filtered (0.45 μm pore size, EZ-Pak, Merck Millipore) to obtain a minimum of 10 and a maximum of 200 colonies. The filter was incubated on the agar for 24 h at 41 °C to minimize background flora. All colonies (phenotypes with and without chromogenic characteristics) were counted to obtain the number of CFU for that selective medium. Colonies were counted according to their morphological distinguishability.

### 2.4 Analysis of environmental bacteria using the protein-based MALDI-TOF MS identification tool

Exemplary isolates from the media as described in the previous section (2.3) were transferred to blood agar (Columbia Agar, 5 % sheep blood) and again incubated at 36 °C for 24 h in order to be identified with MALDI-TOF MS (Bruker) according to manufacturer's instructions. The identified isolates were only documented on a species level, if the score value generated by the BioTyper Software Packet (Version: 4.1.80, Bruker Daltonik) was between 2.0 and 3.0. Otherwise, i.e. with a score value of 1.7–1.99 or different species among the first 10 hits, the identity of the isolates was only noted at the genus level.

### 2.5 Culture-independent analysis of water samples using qPCR

For a culture-independent analysis of ARGs on a molecular level, the concentrations of relevant β-lactam resistance genes were determined by quantitative real-time PCR (qPCR). Therefore, a volume of 500–1000 mL of each water sample was vacuum membrane filtered (0.2 μm Supor®-200 membranes, Pall Life Science) and the total DNA was extracted directly from the membranes by using the FastDNA™ SPIN Kit for soil (MP Biomedicals) according to the manufacturer's instructions with a final elution volume of 100 μL. The investigated genes, the primer sequences and their conferring gene expressions are listed in Table 1. All qPCRs were performed using a Rotor-Gene 6000 cycler (Corbett) with SsoAdvanced Universal SYBR Green Supermix (Bio-Rad). The temperature profile for the amplification was as follows: 2 min 98 °C (initial phase for enzyme activation), 45 cycles of 20 s at 98 °C (denaturation), 20 s at primer specific annealing temperature (T_A_) and fragment length dependent elongation time (t_E_) at 72 °C, followed by melting curve analysis.

**Table 1:** Investigated genes for qPCR analysis, with amplicon length in nucleotides (nt), PCR annealing temperature (T_A_), PCR elongation time (t_E_), enzyme expression and primer sequences forward (F) and reverse (R).

gene	amplicon [nt]	T_A_ [°C]	t_E_ [s]	expression	Primer sequences	Ref.
*bla*_CMY-2_	172	68	20	cephalosporinase	F: CGTTAATCGCACCATCACC R: CGTCTTACTAACCGATCCTAGC	[58]
*bla*_*TEM*_	112	66	20	cephalosporinase	F: TTCCTGTTTTGCTCACCCAG R: CGTCTTACTAACCGATCCTAGC	[59]
*bla*_CTX-M-32_	155	63	20	cephalosporinase	F: CGTCACGCTGTTGTTAGGAA R: CGCTCATCAGCACGATAAAG	[60]
*bla*_SHV_	857	55	30	cephalosporinase/carbapenemase	F: TCGCCTGTGTATTATCTCCC R: CGCAGATAAATCACCACAATG	[61]
*bla*_NDM-1_	154	60	20	cephalosporinase/carbapenemase	F: ATTAGCCGCTGCATTGAT R: CATGTCGAGATAGGAAGTG	[62]
*bla*_OXA-48_	177	65	20	carbapenemase	F: TGTTTTTGGTGGCATCGAT R: GTAAMRATGCTTGGTTCGC	[63]
*bla*_KPC-*3*_	196	69	20	carbapenemase	F: CAGCTCATTCAAGGGCTTTC R: GGCGGCGTTATCACTGTATT	[64]
*bla*_VIM-2_	382	63	20	carbapenemase	F: GTTTGGTCGCATATCGCAAC R: AATGCGCAGCACCAGGATAG	[65]

All samples and standards were analyzed in duplicates. The qPCR standards were prepared from the serial dilutions of known quantities of linearized plasmid containing target genes. For quality control, R^2^ of the standard curve as well as the amplification efficiency were determined and melt curve analysis was performed. Only qPCR experiments with R^2^ values > 0.990 and efficiencies between 90 and 105% were considered. Amplification products were verified via QIAxcel® Advanced system (Qiagen). An overall limit of quantification (LOQ) was 10 copies per qPCR reaction, which for the concentrated samples means a LOQ of about 1 gene copy per mL.

### 2.6 Testing of β-lactamase expression

Selected isolates from R2A-based media were tested for expression of genetically encoded β-lactamase-types, as listed in Table 2, with the Micronaut-S β-Lactamases test (Merlin Diagnostika GmbH), according to manufacturer's instructions. By detecting the growth of a bacterial strain in the presence of a β-lactam antibiotics and an inhibited growth in the presence of the β-lactam and a corresponding β-lactamase inhibitor, the assay can monitor enzymatically mediated resistances. The detectable resistances and tested β-lactamase inhibitors are listed in Table 2.

**Table 2:** Detectable resistances, inhibitors and β-lactamase types with the Micronaut-S β-lactamase assay.

resistance against		inhibition of β-lactamase with			
Cefepim	**CEP**	Clavulanic acid^a^			
Ceftazidim	**CEF**	Clavulanic acid^a^	Avibactam (3-APB)^b^		
Cefotaxim	**CTX**	Clavulanic acid^a^	Avibactam (3-APB)^b^		
Meropenem	**MER**		Avibactam (3-APB)^c^	EDTA^d^	no inhibition
Ertapenem, Temocillin	**ERT, TEM**				no inhibition

no inhibition: possible D carbapenemase producers

a type A cephalosporinase producers

b type C cephalosporinase producers

c type A carbapenemase producers

d type B carbapenemase producers

The following adjustments had to be applied to achieve results for environmental (oligotrophic) isolates: Colonies were grown on R2A medium (Merck) before resuspension in 0.9 % sodium chloride to a turbidity of a McFarland 0.5 standard. After resuspension, 50 μL of each culture were added to the Mueller-Hinton-Broth (provided with the test) and mixed well. Subsequently, 100 μL of the suspension were transferred to each well of the Micronaut-S 96-well plate with the probounded, dried antibiotics and β-lactamase inhibitors. The plate was incubated at 21 °C for 24 h, instead of 36 °C, to allow the growth of the environmental bacteria. After checking the increased turbidity in the growth control (GC), 30 μL Presto Blue Cell Viability agent (Thermo Fisher Scientific) were added to each well and incubated for another 12–24 h until a clear color change from blue to pink was evident. Results were visually and photometrically evaluated by either define the pink wells as “positive” for bacterial growth or measuring the two wavelength 570 nm and 595 nm. For E_570nm_ - E_595nm_ > 0, the colonies were defined as “positive” for bacterial growth as well. The MIC ratio was calculated by dividing the lowest antibiotic concentration that did not allow bacterial growth by lowest antibiotic/inhibitor concentration that did not allow bacterial growth. The expression of a specific β-lactamase was defined as positive for an MIC ratio ≥8.

### 2.7 Data handling and statistical analyses

The collected data were further processed and statistically treated using Microsoft Excel 2016. Ratios between CFU on different media were calculated per sample and the mean value of all ratios was calculated. For resistance gene results < LOQ, “0.0” was used for calculation of mean values.

## 3 Results and discussion

### 3.1 Culture-based detection for β-lactam resistant bacteria

**Fig. 1:**
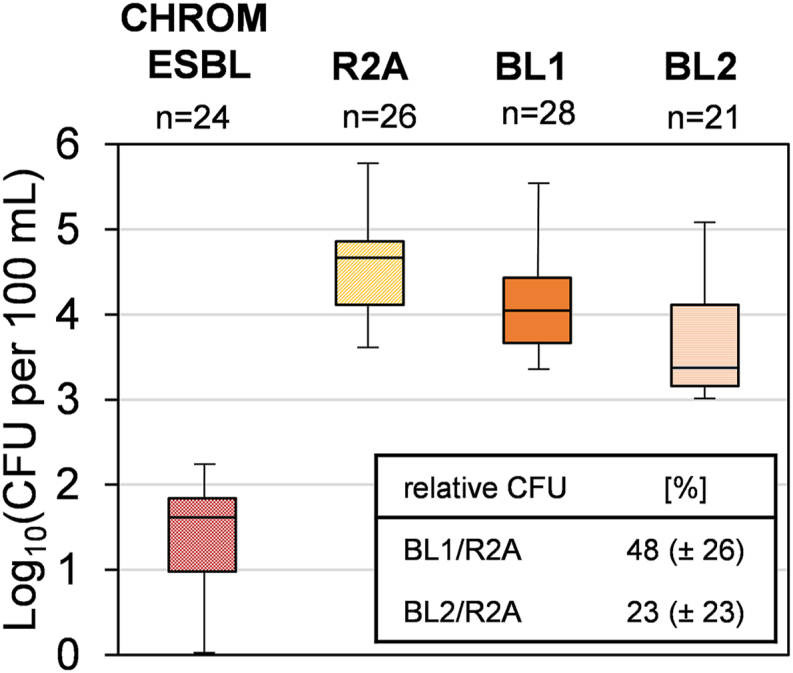
Colony forming units (CFU) for the four tested media and incubation conditions (n is the number of tested samples). On CHROM ESBL the potential pathogenic ESBL-producing bacteria are isolated, on R2A the general oligotrophic bacteria are isolated, on BL1 the 3^rd^ generation cephalosporin resistant bacteria are isolated and on BL2 the carbapenem resistant strains are isolated. See Table S2 (supplementary material) for specific results.

High variances in the oligotrophic and ESBL-producing bacterial numbers were observed in the different rivers, and also in the samples from the same rivers collected at different sampling dates (Fig. 1). The ESBL-producing bacterial numbers on CHROM ESBL medium ranged from 0 to 171 CFU/100 mL. 2 to 3 log_10_ higher numbers of oligotrophic bacteria were detected on the R2A-based and β-lactam-spiked cultivation media. With a median value of 6.1 × 10^4^ CFU/100 mL (minimum 4.0 × 10^3^ CFU/100 mL, maximum 7.6 × 10^5^ CFU/100 mL) the highest colony counts were obtained on the antibiotic free R2A medium. The bacterial numbers were slightly lower on the R2A media, containing cephalosporines and a carbapenem with 1.7 × 10^4^ CFU/100 mL on BL1 and 4.0 × 10^3^ CFU/100 mL on BL2, respectively. All water samples revealed a recurring trend for the presence of β-lactam resistant bacteria, when CFU ratios between media were compared (Fig. 1): An average ratio of 48.3 % (±26.1 %) for BL1/R2A was observed, while the average ratio for the BL2/R2A media was 23 % (±23.0 %). Similar ratios were observed when applying the stamp method (Fig. S1, supplementary material).

The high variation in the occurrence of β-lactam resistant bacteria can be explained by seasonal effects (rain and dry periods), which was also previously reported by Siedlecka et al. (2020) [66]. The range of ESBL bacteria with a median value of 41 CFU/100 mL on the CHROM ESBL agar is consistent with the findings by other researchers who applied the same method in USA and Germany [27,45]. An overview of the findings for antibiotic resistant bacteria with nutrient rich media is summarized in Supplementary Material Table S1. Similar findings have also been reported by Lamba et al. (2020) [67], who traced high ARB and ARGs in the river water back to sewage discharge. However, none of these publications considered any comparison to CFU counts of oligotrophic bacteria in the investigated water samples.

In our study, 4.0 × 10^3^ to 7.6 × 10^5^ CFU per 100 mL were obtained on the non-selective R2A media. In comparison, total heterotrophic counts ranging from 10^5^ and 10^6^ CFU/100 mL on R2A medium have been previously reported from Han River, Korea [52], and 10^6^ CFU/100 mL were reported from Seine River, France [53]. Piotrowska et al. (2017) [54] also reported similar numbers (3.7 × 10^6^ CFU/100 mL) on R2A agar without supplements from an aquatic environmental sample.

Previous studies have already reported the occurrence of antibiotic resistances in oligotrophic environmental bacteria, mainly for often applied antibiotics such as tetracyclines (Table 3). For example, 10^2^–10^6^ CFU/100 mL were obtained with R2A agar supplemented with tetracycline, amoxicillin, and sulfometoxazole for river Seine samples in France [53]. Only few studies are available applying last resort antibiotics [55]. However, in this study also resistances were shown for mixtures of β-lactam antibiotics on the cephalosporine spiked media (BL1, 2.4 × 10^3^ to 5.5 × 10^5^ CFU/100 mL) and carbapenem spiked media (BL2, 1.0 × 10^3^ to 2.0 × 10^5^ CFU/100 mL).

**Table 3:** Comparison of CFU isolated from different surface waters worldwide (water matrix) on R2A media supplemented with antibiotics or without (none) supplements or % (CFU relative to those CFU on R2A without antibiotics, marked with *) in other studies and in this study. Incubation conditions (temperatures and times) are also given.

		Incubation			
water matrix	added antibiotics	temp[°C]	time [d]	CFU per 100 mL or %*	Ref.
river water (River Mahananda, India)	*none*, subsequent susceptibility	30	3	1 × 10^5^ to 5.9 × 10^6^	[56]
Testing					
river water (River Seine, France)	*none*	20	7	1 × 10^6^ to 7 × 10^6^	[53]
	4 mg/L amoxicillin			5 × 10^5^ to 1 × 10^6^	
	50 mg/L amoxicillin			5 × 10^4^ to 7 × 10^5^	
	4 mg/L tetracycline			2 × 10^4^ to 5 × 10^5^	
	300 mg/L tetracycline			2 × 10^2^ to 5 × 10^3^	
	16 mg/L sulfomethaxole			8 × 10^4^ to 1 × 10^6^	
	300 mg/L sulfomethaxole			8 × 10^3^ to 5 × 10^5^	
river water (River Han, South Korea)	*none*	25	3	3.4 × 10^5^ to 1.1 × 10^6^	[52]
	100 mg/L vancomycin			6.1 × 10^4^ to 5.5 × 10^5^	
pond water from *Cyprinus carpio* farms (Poland)	*none*	room-	1 to 2	3.7 × 10^6^	[54]
	10 mg/L streptomycin	temp.		7.0 × 10^4^	
	10 mg/L tetracycline			2.8 × 10^5^	
	10 mg/L erythromycin			2.1 × 10^4^	
tap water (Poland)	*none*	22	7	4 × 10^2^ to 2.8 × 10^3^	[57]
	8 mg/L ceftazidim			40.4–99.9 %*	
	8 mg/L amoxicillin			0.0–3.8 %*	
	8 mg/L ciprofloxacin			0.0–0.02 %*	
	16 mg/L tetracycline			0.0–1.2 %*	
biofilms from river water	*none*	22	7	7.6 × 10^3^ to 1.1 × 10^6^	[55]
(River Rhine, Germany)	32 mg/L ceftazidim			11 (±1.6) %*	
	32 mg/L cefazoline			8.1 (±0.0) %*	
	16 mg/L penicillin G			31 (±3.3) %*	
	32 mg/L vancomycin			2.3 (±0.5) %*	
	8 mg/L imipenem			0.4 (±0.1) %*	
river water (River Rhine, Danube, Ruhr, Stever)	*none*	21	2	4.0 × 10^3^ to 7.6 × 10^5^	this study
	3^rd^ generation cephalosporines			2.4 × 10^3^ to 5.5 × 10^5^	
	−2 mg/L cefotaxim			or 48.3 (±26.1) %*	
	−2 mg/L ceftazidim				
	−4 mg/L cefpodoxim				
	carbapenem & cephalosporine			1.0 × 10^3^ to 2.0 × 10^5^	
	−2 mg/L meropenem			or 22.5 (±23.0) %*	
	−4 mg/L ceftazidim				

The prescription of carbapenem antibiotics in human and veterinary medicine is restricted in Germany since they are categorized as last-resort antibiotics. The application of carbapenems is significantly lower compared to 3^rd^ and 4^th^ generation cephalosporines in German hospitals [68]. In contrast to that, GERMAP [37] reported a considerably increased application of oral cephalosporines and it was also shown, that the resistance against carbapenems still plays a subordinate role among infectious *E. coli* isolated from hospital settings (<1 %). Therefore, carbapenems (like meropenem) still have great importance in treating pathogens resistant to more commonly applied antibiotics. This is for example valid for *E. coli* with an ESBL phenotype or fluorochinolin resistance, which have been significantly increasing between 1995 and 2010 [37]. Since a higher application of cephalosporines compared to carbapenems is accompanied with a higher ratio of cephalosporine resistant ARB compared to carbapenem resistant ARB, a correlation between high application and a faster promotion of AMR development and spread in the environment could be assumed.

### 3.2 Identification of potential pathogenic and environmental isolates

**Fig. 2:**
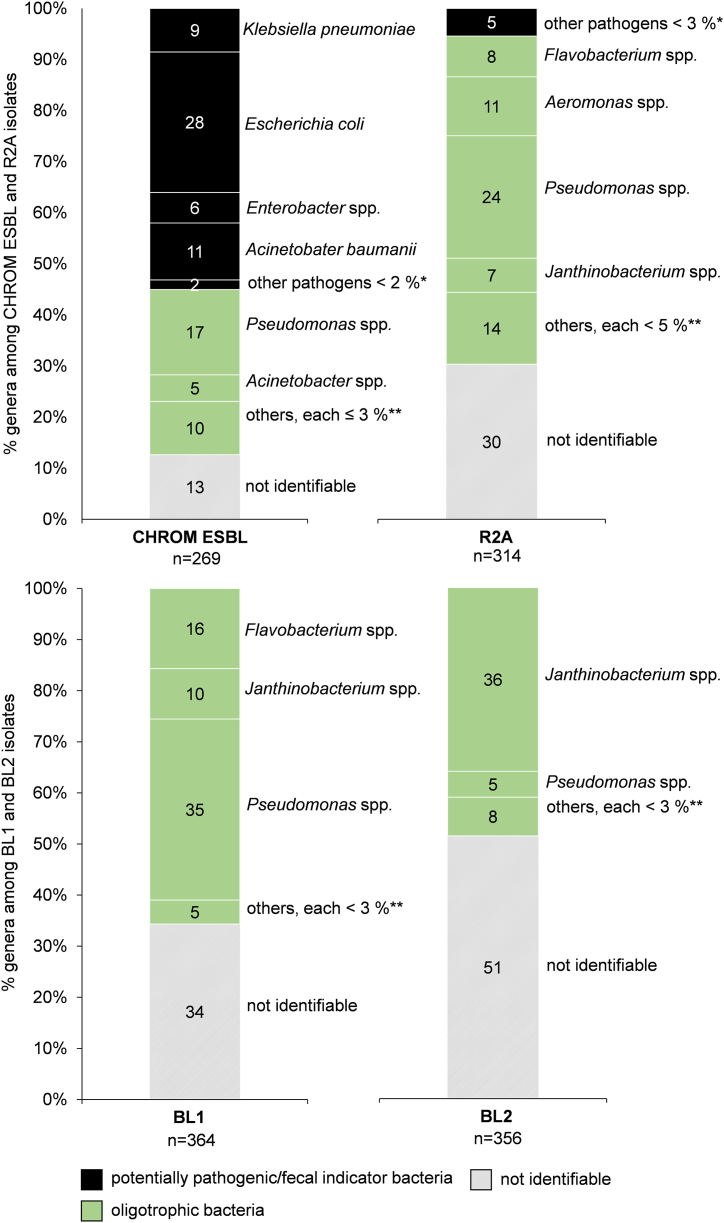
Bacterial isolates analyzed with MALDI-TOF MS and the resulting categorization in “potentially pathogenic/fecal indicator bacteria” (black colored, acc. to World Health Organization (2017) [33] and RKI (2021) [50]), “oligotrophic” (green colored) and “not identifiable” (with no result from MALDI-TOF MS, grey colored/stripes) on CHROM ESBL and R2A-based media with and without antibiotic supplements. “n” is the total number of analyzed isolates (all investigated water samples included). Other pathogens (*) and other oligotrophic bacteria (**), below 2, 3 or 5 % each, are summarized in Table S3. (For interpretation of the references to color in this figure legend, the reader is referred to the Web version of this article.)

The application of MALDI-TOF MS allowed rapid identification of many isolates which grew on the CHROM ESBL and the R2A-based media with and without β-lactam antibiotics (Fig. 2). The relationship between the three groups (oligotrophic, potentially pathogenic/fecal indicator bacteria and not identifiable) among 269 isolates used for MALDI-TOF MS identification from CHROM ESBL medium in total, is different from those found among the isolates grown on R2A-based media. A total of 55 % of our isolates could be clearly identified as potential pathogens and fecal indicator bacteria, as per WHO classification. ESBL-producing *E. coli* were predominantly detected (27.5 %) among all CHROM ESBL isolates. Almost half of the total isolates (45 %) did not belong to the clinically relevant group, with 13 % of isolates unidentifiable by MALDI-TOF MS. The identified non-pathogenic strains were from genera such as *Acinetobacter*, *Pseudomonas* and *Bordatella* (various species) Previously, in Germany, *Bordatella* strains isolated from swine, dogs, and cats with respiratory diseases showed dramatically high minimal inhibitory concentration (MIC) values for multiple β-lactams [37]. CHROM ESBL medium has been previously used to isolate pathogenic and fecal indicator strains such as *Acinetobacter baumanii, E*. *coli, Pseudomonas aeruginosa, Klebsiella pneumoniae, Enterobacter* spp.*, Shigella* spp. and *Serratia* spp. [27].

Among oligotrophic bacteria isolated on the antibiotic-free R2A media, strains belonging to the genus *Pseudomonas* (24 %) were more common followed by the *Aeromonas*, *Flavobacterium,* and *Janthinobacterium* genera with 26 %. All other genera were less prevalent (<5 %) including potential pathogens such as *Klebsiella* (0.3 %) and *Enterobacter* (2.5 %) (see Table S3, supplementary material). The MALDI-TOF MS identification of 364 isolates grown on BL1 and 356 isolates grown on BL2 did not reveal any pathogenic or fecal indicator strain. On BL1, *Pseudomonas, Flavobacterium,* and *Janthinobacterium* were most frequently identified with 35 %, 16 %, and 10 %, respectively. Oligotrophic bacteria, including Ba*cillus, Chryseobacterium, Flavobacterium, Pseudomonas,* *Stenotrophomonas*, *Paenibacillus* and *Sphingobacterium,* have been previously isolated from fish ponds on R2A agar with streptomycin, tetracycline, or erythromycin spiked R2A media [54]. The results of this study demonstrate, that oligotrophic bacteria, carrying a variety of ARGs, are detected in environmental habitats influenced by anthropogenic activities. These bacteria obviously are reservoirs for several types of ARGs. In a study from Italy, the expression of metallo-β-lactamases in soil isolates of the genera *Aeromonas, Chryseobacterium, Janthinobacterium* and *Stenotrophomonas* has been already reported [69]. An environmental *Stenotrophomonas* strain with chromosome-borne resistance to β-lactam was shown to be highly resistant to meropenem [70]. Narciso-da-Rocha and Manaia (2016) [71] also reported *a* widespread prevalence of multi-drug resistance in the environment, including β-lactam-resistant *Aeromonas, Chryseobacterium, Janthinobacterium,* and *Stenotrophomonas*. It has also been reported, that resistance genes in strains like *K. pneumoniae, E. coli,* or *Enterobacter* sp. have been transferred from the oligotrophic and non-pathogenic strains such as *Sphingobacterium* or *Shewanella* [11]. The mentioned studies were using culturing and enzyme testing methods [69] or rather *16S* rRNA gene sequencing methods [54,70,71]. Therefore, to our knowledge, our study is the first analyzing environmental bacteria using the protein-based MALDI-TOF MS identification tool, which has the advantage to have a comparably high specificity but is less time consuming, compared to *16S* rRNA gene sequencing.

MALDI-TOF MS is emerging as a rapid and cost-effective method for the identification of bacterial isolates [72]. There is still a lack of protein spectra in the database when comparing the efficiency for identification with DNA-sequencing methods, in particular for environmental isolates. However, the method can be used to identify clinically relevant bacteria fast and quite reliably in environmental water habitats.

### 3.3 β-lactam resistance genes in surface waters

**Fig. 3:**
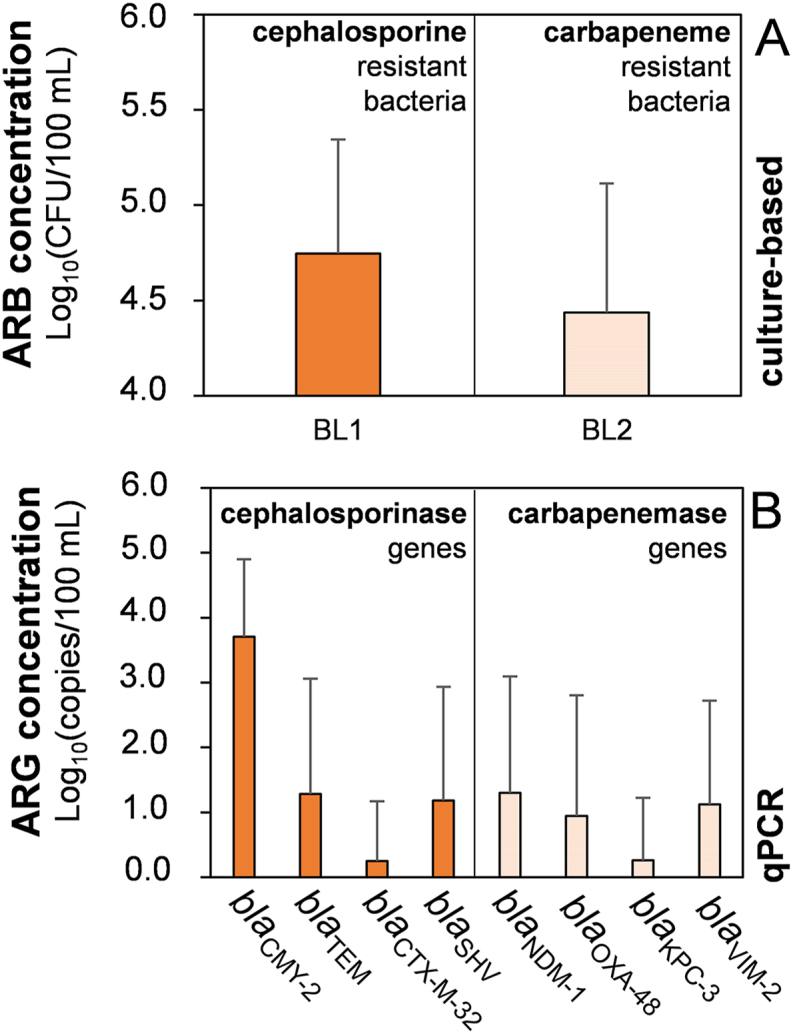
Comparison between culture-based and qPCR-based methods for detecting antibiotic resistances in the environment. Shown are the absolute concentrations of cephalosporin (plain) and carbapenem (stripes) resistant bacteria (A) and resistance genes (B).

Multiple clinically relevant cephalosporine (cephalosporinase genes), and carbapenem resistance genes (carbapenemase genes) were analyzed in 28 surface water samples. The results for absolute gene copy numbers per 100 mL sample are shown in Table S4 (supplementary material). The cephalosporinase genes *bla*_TEM_ and *bla*_CMY-2_ were found in the highest concentrations ranging from 1.3 to 3.7 log_10_ copies/100 mL in all tested samples, while carbapenemase genes like *bla*_OXA-48_ and *bla*_KPC-3_ were present in only 2 to 6 samples, at lower concentrations of 1.0 and 0.3 log copies/100 mL, respectively (Fig. 3, B). Also, *bla*_VIM-2_ and *bla*_NDM-1_ genes were detected in intermediary frequency in the surface water samples with an average concentration of 1.1 and 1.3 log_10_ copies/100 mL, respectively. The cephalosporinase gene *bla*_CTX-M-32_ was detected at a much lower concentration of 0.3 log_10_ copies/100 mL. All ARGs are categorized as relevant in clinical strains and have been detected in rivers all over the world [8,67,[73], [74], [75], [76], [77], [78]]. They were also detected in at least one, but more often in more than 20 or 30 % of the surface water samples in our study.

A clear comparable trend can be seen between the occurrence of the resistance genes and the grown oligotrophic bacteria on the antibiotic-containing R2A media BL1 and BL2 (Fig. 3A and B). The resistance genes, coding only for cephalosporine resistance, and the cephalosporine-resistant oligotrophs occur in increased concentrations, compared to the carbapenemase genes and carbapenem-resistant bacteria. The chosen culture-based method with an incubation time of only two days allowed a very high throughput and a good overview of viable resistant strains. Nonetheless, some environmental bacteria might grow much slower and are therefore still undetectable with this approach [54]. Therefore, the application of PCR-based methods is recommended to obtain a more detailed picture of the total prevalence of ARGs in the environmental samples.

A high prevalence of β-lactam and cephalosporine resistance genes (almost 100 %) was also reported in river water samples from India, whereas the prevalence of carbapenem resistance was significantly low (10–20 %) [79]. Müller et al. (2018) [80] reported the presence of the *bla*_VIM_ resistance gene in surface waters, whereas *bla*_KPC_, *bla*_OXA-48_ and *bla*_NDM_ were only found in isolates of clinical/urban wastewaters. However, *bla*_KPC_, *bla*_OXA-48_ and *bla*_NDM_ genes were present more widely in the surface waters investigated in our study.

### 3.4 Phenotypic detection of β-lactamases in oligotrophic isolates

Recent studies have successfully applied the Micronaut-S test for detecting β-lactamases on bacterial isolates such as *Escherichia*, *Enterobacter*, *Pseudomonas* and *Klebsiella* from fish ponds, dairy farms, and broiler chicken farms [81,82]. However, to our knowledge, this is the first study that demonstrates the application for surface water isolates. A total of 37 isolates from the BL2 plates were used for the Micronaut-S β-Lactamases test. As evident from the results of the Micronaut-S tests, some isolates were able to grow in presence of the antibiotics as well as in presence of the antibiotic/inhibitor combination. The resistance to antibiotic/inhibitor combinations has been earlier reported for *E. coli* and *Klebsiella oxytoca* clinical isolates, where they were attributed to excessive production of type A lactamases, such as TEM-1 or SHV-1 [[83], [84], [85]], and/or to altered permeability of the outer membrane [86].

From one BL2 plate (representing one sample), all isolates (n=13) were examined in the Micronaut-S assay and were all found to express β-lactamases. Many isolates showed β-lactamase mediated resistances to multiple antibiotics (Table 4). Among detected ARGs, the clavulanic acid-inhibited cefepim (CEP), ceftazidim (CEF), or cefotaxim (CTX) resistance (showing type A cephalosporinase production) were most frequently detected in up to 22 isolates. Additionally, four isolates expressed Avibactam inhibitable β-lactamases, conferring resistance to CEF and/or CTX (showing type C cephalosporinase production), respectively. Seven isolates out of a total of 37 isolates (19%), showed resistance to meropenem, and β-lactamase expression could be reliably proven, as the growth of the isolates was inhibited by either Avibactam or EDTA (A and B carbapenemases). For the rest of the 30 isolates (81 %) resistant to meropenem (MER) and/or ertapenem (ERT) and temocillin (TEM), no inhibition with avibactam or EDTA was detected. For clinical isolates, these results suggest D carbapenemase production (as per manufacturer's instructions of the Micronaut-S test). However, for environmental isolates, other resistance mechanisms, such as target modification mechanisms like penicillin-binding proteins (PBPs) or efflux pumps [87] could also explain the insensitivity against the tested carbapenems. Lipid production in bacterial cells is known to be upregulated to stabilize the cell wall in the presence of β-lactams [88]. This could also explain observed insensitivity in these isolates.

**Table 4:** Resistances and β-lactamase inhibitions according to the Micronaut-S test evaluated from a total of n=37 picked isolates from BL2 plates.

	CEP	CEF	CTX	MER	ERT, TEM	negative
**N**^**o**^**. of resistant BL2 isolates:**	22	30	31	37	37	0
**N**^**o**^**. of isolates where growth was inhibited with**						
clavulanic acid:	15	21	22	–	–	–
Avibactam:	–	4	4	3	–	–
EDTA:	–	–	–	4	–	–
**N**^**o**^**. of isolates showing no inhibition with**						
avibactam or EDTA:				30	30	–

CEP = cefepim, CEF = ceftazidim, CTX = cefotaxim, MER = meropenem, ERT = ertapenem, TEM = temocillin.

Isolates (n=7) from genera *Erwinia, Enterobacter, Lelliottia, Pantoea, Shewanella, Janthinobacterium,* and *Yersinia* selected from antibiotic-free R2A-medium were additionally tested with the Micronaut-S test. Two isolates (*Yersinia* and *Pantoea* strains) were completely sensitive against all tested β-lactams (see Table S5, supplementary material). An *Enterobacter* isolate showed resistance against all tested antibiotics (CEP, CEF, CTX, MER, ERT, and TEM). Leister and Hügler (2022) [32] had previously reported intrinsic resistance to β-lactams in *Enterobacter*, and in *Lelliottia* species isolated from drinking water reservoirs. We have also found expression of type A carbapenemases in our isolates from the *Lelliottia* species.

Intrinsic resistance to several antibiotic classes has already been described in the genome of *Elizabethkingia anopheles* [89] and reviewed for several other environmental bacteria by Grenni et al. (2018) [90]. Among them, some β-lactamases such as metallo β-lactamases (MBLs, type B carbapenemases) and PBPs have been detected by genome annotation [91]. These PBPs are associated with the induction of AmpC β-lactamases (type C cephalosporinases) [92]. Phylogenetic analyses of selected β-lactamases in *E. anopheles* strains suggest that certain β-lactamase genes were not only vertically inherited but also acquired by lateral gene transfer [91]. Type C (includes ESBL, AmpC) and D (includes e.g. OXA-48) β-lactamases have been identified in the environmental isolates such as *Duganella* sp.*, Janthinobacterium lividum, and Massilia* sp. in a study by Silveira et al. (2018) [93]. Functional metallo-β-lactamase genes (type B carbapenemases) have already been described in *Janthinobacterium lividum* and *Massilia oculi* [94,95] and were shown to be phylogenetically related to acquired β-lactamases produced by clinical pathogens. According to Gudeta et al. (2016) [95], these may have been acquired from members of the *Oxalobacteraceae* family.

By analyzing genomes of 76 *Herbaspirillium* isolates (from clinical and environmental settings), Oliveira et al. (2021) [96] demonstrated that type A, B, C, and D β-lactamases are also common in this genus, although the genes for type C and D enzymes were only found in isolates that did not encode for type A and B β-lactamases. Isolates of the genus *Pedobacter* were found to be positive for ESBL (type A cephalosporinases) and OXA-48 (type D carbapenemases). This is in agreement with the previously reported carbapenems and cephalosporines resistance of *Pedobacter* isolates [97]. A high rate of β-lactam resistance, including to cephalosporines (>82 % of 125 isolates) among *Pseudomonas* sp. Isolates, has been reported from Nigeria [98]. Since 50 % of these *Pseudomonas* sp. isolates contained 2 to 3 plasmids, it was hypothesized that *Pseudomonas* sp. may play an important role in the spread of plasmids and possibly AMR in the aquatic environment.

## 4 Conclusion

A culture-based method (based on R2A medium, containing cephalosporines and a carbapenem) was successfully developed and applied to isolate β-lactamase producing oligotrophic bacteria from German River waters. It was observed that β-lactam resistances, highly relevant in clinical pathogens, are already widespread among environmental bacteria, with CFUs factor 100 to 1000 higher than ESBL producing pathogens and indicator organisms. Resistances against carbapenems were detected in lower frequency (23 %) compared to cephalosporine resistances (48 %). A subsequent characterization of bacterial genera with MALDI-TOF MS revealed, that isolates belonged to environmental genera such as *Pseudomonas, Janthinobacterium* and *Flavobacterium*. Some ARB with a lower MIC might not be detected with the applied antibiotic concentration but still, the presented culture-based method gives a fast and clear insight to the quantity of oligotrophic bacteria with highly relevant resistances in an environmental sample. In particular, clinical relevant ESBL bacteria can be reliably identified with MALDI-TOF MS. The post-culturing Micronaut-S method was shown to be applicable for environmental isolates under adapted incubation conditions. For all tested isolates from selective media (BL2) it could proof the production and activity of β-lactamases, with type A cephalosporinases (78 % of the tested isolates) and carbapenemases to be the predominant enzymes.

The findings of this study emphasize the need to investigate not only resistant pathogens, but also resistant oligotrophic bacteria in the aquatic environment, as they pose a risk to human health in terms of uncontrollable AMR transmission. As the culture-based method is practical and cheap, it can be easily redesigned and applied also in other regions, by simply adding other antibiotics to R2A agar, to analyze other resistant environmental bacteria of interest. This can be of special interest for new generation and last-resort antibiotics.

## Funding statement

The authors gratefully acknowledge funding by German Technical and Scientific Association for Gas and Water (DVGW) [W2018/30].

## Data availability statement

Data will be made available on request.

## CRediT authorship contribution statement

**Lara Stelmaszyk:** Writing – review & editing, Writing – original draft, Visualization, Methodology, Investigation, Formal analysis, Data curation, Conceptualization. **Claudia Stange:** Validation, Methodology, Funding acquisition, Formal analysis, Conceptualization. **Michael Hügler:** Writing – review & editing, Writing – original draft, Software, Methodology, Investigation. **Jatinder P.S. Sidhu:** Writing – original draft, Validation, Methodology, Funding acquisition, Conceptualization. **Harald Horn:** Writing – original draft, Visualization, Supervision, Methodology, Data curation, Conceptualization. **Andreas Tiehm:** Writing – review & editing, Writing – original draft, Validation, Supervision, Methodology, Funding acquisition, Formal analysis, Data curation, Conceptualization.

## Declaration of competing interest

The authors declare that they have no known competing financial interests or personal relationships that could have appeared to influence the work reported in this paper.
