# Supplementary material for: Quantification of β-lactamase producing bacteria in German surface waters with subsequent MALDI-TOF MS-based identification and β-lactamase activity assay
Source: Heliyon. 2024 Mar 5;10(5):e27384. doi: 10.1016/j.heliyon.2024.e27384 (PMC10937694; doi:10.1016/j.heliyon.2024.e27384)
Supplement: Supplementary Material [file mmc1.docx]

Quantification of β-lactamase producing bacteria in German surface waters with subsequent MALDI-TOF MS-based identification and β-lactamase activity assay

Lara Stelmaszyk^1^*, Claudia Stange^1^, Michael Hügler^1^, Jatinder P.S. Sidhu^2^, Harald Horn^3^, Andreas Tiehm^1^

^1^TZW: DVGW Technologiezentrum Wasser, Department of Water Microbiology, Karlsruher Straße 84, Karlsruhe, Germany

^2^CSIRO Oceans and Atmosphere, Ecosciences Precinct, 41 Boggo Road, Brisbane, Australia

**^3^**Karlsruher Institut für Technologie, Engler-Bunte Institute, Wasserchemie und Wassertechnologie, Karlsruhe, Germany

*****Correspondence: lara.stelmaszyk@tzw.de

# Supplementary information

**Supplementary Table S1** Comparison of CFU isolated on nutrient-rich culturing media supplemented with β-lactam antibiotics or % (ESBL bacteria per investigated isolates) in other studies and in this study. All CFU-values are related to the volume of 100 mL for better comparison. Incubation was performed at 35 to 41 °C for 24 or 48 hours in every study (Haberecht et al., 2019).

| **References** | **Water matrix** | **Added antibiotics** | **CFU per 100 mL or %*** |
| --- | --- | --- | --- |
|  |  |  |  |
|  |  |  |  |
|  |  |  |  |
| Adelowo et al., 2018 | rivers (Nigeria) | 6 mg/L ceftazidime | 7.9 % |
|  |  | 6 mg/L cefpodoxime |  |
| Caltagirone *et al.* 2017 | river water (Italy) | 8 mg/L cefotaxime | 2.8x10^5^ to 3.71x10^5^ |
|  |  |  | or 6.4 to 18.8 % |
| Diab et al., 2018 | spring water, estuaries (Lebanon) | 4 mg/L ceftazidime | *no quantification* |
|  |  | 2 mg/L meropeneme |  |
| Gekenidis et al., 2018 | irrigation water (Switzerland) | 8 mg/L ceftazidime | < 10 to > 3×10^2^ (only *E. coli*) |
|  |  | *unknown* (Oxoid Brilliance ESBL) | < 10 to > 3×10^2^ (only *E. coli*) |
|  |  |  |  |
|  |  |  |  |
| Haberecht et al., 2019 | surface waters (Colorado) | unknown (CHROMagar^TM^ ESBL) | 1.4x10^3^ (±1.9x10^3^) |
|  |  | unknown (CHROMagar^TM^ KPC) | 5.8x10^2^ (±5.5x10^2^) |
| Korzeniewska et al., 2013 | river water (Łyna River, Poland) | none, subsequent susceptibility | 21.1 to 84.2 % (only *E. coli*) |
|  |  | testing (CTX, CAZ, CPD-disks) |  |
| Schreiber et al., 2021 | surface waters (Germany) | unknown (CHROMagar^TM^ ESBL) | < 1.0x10^2^ to 1x10^3^ |
| this study | river water (River Rhine, Danube, | unknown (CHROMagar^TM^ ESBL) | < 1.0 to 1.7x10^2^ |
|  | Ruhr; Stever) |  |  |
|  | *ESBL bacteria per investigated isolates | | |

**Supplementary Table S2** Results of the culture-based methods. n.i.= not investigated, n.c.= not calculated.

|  |  | **CFU/100 mL** | | | | **relative [%]** | |
| --- | --- | --- | --- | --- | --- | --- | --- |
|  |  | **ChromESBL** | **R2A w/o AB** | **BL1** | **BL2** | **BL1/R2A** | **BL2/BL1** |
| Rhine river | 02.12.2019 | 15 | 36000 | 20000 | 3000 | 56 | 15 |
|  | 27.01.2020 | 66 | 14000 | 4000 | n.i. | 29 | n.c. |
|  | 10.08.2020 | n.i. | 11625 | 2438 | 2438 | 21 | 100 |
|  | 18.01.2021 | 171 | 381000 | 300000 | 124000 | 79 | 41 |
| Ruhr river | 08.10.2019 | 11 | n.i. | 13800 | n.i. | n.c. | n.c. |
|  | 15.11.2019 | 11 | 4000 | 4545 | n.i. | 114 | n.c. |
|  | 02.12.2019 | n.i. | 46000 | 39000 | 20000 | 85 | 51 |
|  | 08.01.2020 | 0.42 | 847 | 267 | n.i. | 31 | n.c. |
|  | 05.02.2020 | 101 | 8182 | 3100 | 2800 | 38 | 90 |
|  | 07.04.2020 | 117 | 120000 | 92000 | 22000 | 77 | 24 |
|  | 05.05.2020 | 3.6 | 64000 | 14000 | 4000 | 22 | 29 |
|  | 09.06.2020 | 0.9 | 64000 | 14000 | 2000 | 22 | 14 |
|  | 04.02.2021 | 43 | 282467 | 144433 | 34292 | 51 | 24 |
| Stever river | 08.10.2019 | 26 | n.i. | 9636 | n.i. | n.c. | n.c. |
|  | 15.11.2019 | 3.6 | 5818 | 3545 | n.i. | 61 | n.c. |
|  | 02.12.2019 | n.i. | 16091 | 10455 | 10545 | 65 | 101 |
|  | 08.01.2020 | 1 | 606 | 469 | n.u. | 77 | n.c. |
|  | 05.02.2020 | 35 | 10182 | 3818 | 3091 | 38 | 81 |
|  | 07.04.2020 | 63 | 258000 | 30000 | 3000 | 12 | 10 |
|  | 05.05.2020 | 58 | 168000 | 50000 | 12000 | 30 | 24 |
|  | 09.06.2020 | 0.00 | 86000 | 28000 | 4000 | 33 | 14 |
|  | 04.02.2021 | 54 | 759333 | 546667 | 202000 | 72 | 37 |
| Danube river | 17.02.2020 | 80 | 66000 | 31000 | 39000 | 47 | 126 |
|  | 07.04.2020 | n.i. | 58000 | 7400 | 1700 | 13 | 23 |
|  | 16.06.2020 | 136 | 98000 | 74667 | 78667 | 76 | 105 |
|  | 25.08.2020 | 5.5 | 27667 | 6167 | 2000 | 22 | 32 |
|  | 20.10.2020 | 41 | 61385 | 22188 | 2063 | 36 | 9.3 |
|  | 01.12.2020 | 1.8 | 19667 | 10333 | 1000 | 53 | 10 |

**Supplementary Figure S1** CFU obtained on the R2A-based agar plates with the stemple technique. A 20-fold dilution of the original sample (SW3-Ru) was plated on the original R2A plate without AB supplements.

**Supplementary Table S3** Addition to Figure 2: Other pathogens (P) and other non-pathogenic (NP) /oligotrophic bacteria (O) below 5 % each, among the identified isolates from the four investigated media CHROM ESBL, R2A, BL1 and BL2.

| **others (<5% each)** | | | |
| --- | --- | --- | --- |
| **CHROM ESBL** | | | |
| *Achromobacter* | 2,6 | % | NP |
| *Bordetella* | 0,4 | % | NP |
| *Citrobacter* | 3,0 | % | NP |
| *Pantoea* | 0,7 | % | NP |
| *Ralstonia* | 1,5 | % | NP |
| *Stenotrophomonas* | 2,2 | % | NP |
| *Enterobacter* spp. | 5,9 | % | P |
| *Pseudomonas aeruginosa* | 0,7 | % | P |
| *Shigella spp.* | 1,1 | % | P |
| **R2A** | | | |
| *Acinetobacter* | 4,5 | % | O |
| *Bacillus* | 1,3 | % | O |
| *Chryseobacterium* | 1,0 | % | O |
| *Citrobacter* | 0,3 | % | O |
| *Erwinia* | 0,6 | % | O |
| *Ewingella* | 0,3 | % | O |
| *Exiguobacterium* | 0,3 | % | O |
| *Lelliottia* | 0,6 | % | O |
| *Pseudoescherichia* | 0,3 | % | O |
| *Raoultella* | 1,0 | % | O |
| *Rhanella* | 1,6 | % | O |
| *Shewanella* | 1,6 | % | O |
| *Yersinia* | 0,6 | % | O |
| *Enterobacter* | 2,6 | % | P |
| *Escherichia coli* | 0,3 | % | P |
| *Klebsiella* | 0,3 | % | P |
| *Serratia* | 2,2 | % | P |
| **BL1** | | | |
| *Achromobacter* | 0,3 | % | O |
| *Acinetobacter* | 0,5 | % | O |
| *Bacillus* | 2,5 | % | O |
| *Chryseobacterium* | 0,3 | % | O |
| *Comamonas* | 0,3 | % | O |
| *Paenibacillus* | 0,3 | % | O |
| *Rhanella* | 0,3 | % | O |
| *Stenotrophomonas* | 0,3 | % | O |
| **BL2** | | | |
| *Acinetobacter* | 0,8 | % | O |
| *Bacillus* | 0,6 | % | O |
| *Elizabethkingia* | 1,1 | % | O |
| *Flavobacterium* | 3,1 | % | O |
| *Glutamicibacter* | 0,6 | % | O |
| *Pseudoarthrobacter* | 0,6 | % | O |
| *Sphingobacterium* | 0,3 | % | O |
| *Stenotrophomonas* | 0,3 | % | O |
| *Wautersiella* | 0,3 | % | O |

**Supplementary Table S4** Results of the qPCR measurements of 16S rDNA, intl1 and ARGs. LOQ.= Limit of quantification (100 gene copies per 100 mL), n.a.= not analysed.

|  |  | **gene copies/100 mL** | | | | | | | | | |
| --- | --- | --- | --- | --- | --- | --- | --- | --- | --- | --- | --- |
|  | **date** | ***16S*** | ***intl1*** | ***bla*_CMY-2_** | ***bla*_TEM_** | ***bla*_CTXM-32_** | ***bla*_SHV_** | ***bla*_NDM-1_** | ***bla*_OXA-48_** | ***bla*_KPC-3_** | ***bla*_VIM-2_** |
| Rhine river | 02.12.2019 | 1.2E+08 | 6.9E+05 | 1.7E+04 | < LOQ | < LOQ | < LOQ | < LOQ | < LOQ | < LOQ | < LOQ |
|  | 27.01.2020 | 4.1E+07 | 9.9E+04 | 5.6E+03 | < LOQ | < LOQ | < LOQ | < LOQ | < LOQ | < LOQ | < LOQ |
|  | 10.08.2020 | 2.0E+08 | 6.6E+05 | 4.3E+03 | 5.9E+02 | < LOQ | < LOQ | < LOQ | < LOQ | < LOQ | n.a. |
|  | 18.01.2021 | 1.5E+09 | 1.0E+06 | 1.7E+04 | 2.8E+03 | 4.3E+03 | < LOQ | < LOQ | 1.8E+05 | < LOQ | < LOQ |
| Ruhr river | 08.10.2019 | 2.3E+08 | 4.7E+06 | 6.3E+03 | < LOQ | < LOQ | < LOQ | 1.1E+04 | < LOQ | < LOQ | < LOQ |
|  | 15.11.2019 | 5.7E+06 | 3.2E+04 | 5.3E+02 | < LOQ | < LOQ | < LOQ | < LOQ | < LOQ | < LOQ | < LOQ |
|  | 02.12.2019 | 2.4E+08 | 2.0E+06 | 5.0E+04 | 5.9E+03 | < LOQ | 5.3E+03 | 3.6E+03 | < LOQ | < LOQ | 4.7E+03 |
|  | 08.01.2020 | 2.0E+08 | 1.9E+06 | 5.1E+04 | 7.3E+03 | < LOQ | < LOQ | 4.6E+03 | < LOQ | < LOQ | 2.4E+03 |
|  | 05.02.2020 | 3.0E+08 | 4.3E+06 | 5.1E+04 | 1.2E+04 | 2.4E+03 | 5.7E+03 | 4.7E+03 | < LOQ | < LOQ | < LOQ |
|  | 07.04.2020 | 2.5E+08 | 6.7E+06 | < LOQ | < LOQ | < LOQ | < LOQ | < LOQ | < LOQ | < LOQ | n.a. |
|  | 05.05.2020 | 3.5E+08 | 6.5E+06 | 3.8E+03 | < LOQ | < LOQ | < LOQ | < LOQ | < LOQ | < LOQ | n.a. |
|  | 09.06.2020 | 1.4E+07 | 2.5E+05 | 2.4E+03 | < LOQ | < LOQ | < LOQ | < LOQ | < LOQ | < LOQ | n.a. |
|  | 04.02.2020 | 1.2E+09 | 9.7E+06 | 1.7E+04 | 1.1E+03 | < LOQ | < LOQ | < LOQ | < LOQ | < LOQ | n.a. |
| Stever river | 08.10.2019 | 2.3E+08 | 5.7E+06 | 6.5E+03 | < LOQ | < LOQ | < LOQ | 8.9E+03 | < LOQ | < LOQ | < LOQ |
|  | 15.11.2019 | 4.2E+07 | 1.0E+05 | 1.6E+04 | < LOQ | < LOQ | < LOQ | < LOQ | < LOQ | < LOQ | 3.1E+02 |
|  | 02.12.2019 | 2.7E+08 | 2.3E+06 | 2.5E+04 | < LOQ | < LOQ | 6.6E+03 | 3.2E+03 | < LOQ | < LOQ | 2.9E+03 |
|  | 08.01.2020 | 2.7E+08 | 2.5E+06 | 3.7E+04 | 2.3E+04 | < LOQ | 2.6E+03 | 7.5E+03 | < LOQ | < LOQ | 5.0E+02 |
|  | 05.02.2020 | 2.8E+08 | 2.4E+06 | 5.1E+04 | 5.0E+03 | < LOQ | 2.1E+03 | 5.0E+03 | < LOQ | < LOQ | 2.6E+03 |
|  | 07.04.2020 | 6.1E+08 | 5.3E+06 | < LOQ | < LOQ | < LOQ | < LOQ | < LOQ | < LOQ | < LOQ | n.a. |
|  | 05.05.2020 | 8.0E+08 | 7.0E+06 | 5.1E+03 | < LOQ | < LOQ | < LOQ | < LOQ | < LOQ | < LOQ | n.a. |
|  | 09.06.2020 | 4.4E+07 | 3.5E+05 | 5.9E+03 | < LOQ | < LOQ | < LOQ | < LOQ | < LOQ | < LOQ | n.a. |
|  | 04.02.2020 | 2.9E+09 | 5.0E+06 | 1.8E+04 | 9.9E+02 | < LOQ | < LOQ | < LOQ | < LOQ | 8.2E+03 | n.a. |
| Danube river | 17.02.2020 | 1.2E+08 | 1.5E+06 | 2.8E+04 | < LOQ | < LOQ | 8.8E+03 | 6.0E+03 | < LOQ | < LOQ | < LOQ |
|  | 07.04.2020 | 2.1E+08 | 1.2E+06 | 1.1E+04 | < LOQ | < LOQ | 1.9E+04 | < LOQ | < LOQ | < LOQ | n.a. |
|  | 16.06.2020 | 1.1E+08 | 8.3E+05 | 1.3E+04 | 8.9E+02 | < LOQ | 2.0E+03 | 5.8E+02 | < LOQ | < LOQ | n.a. |
|  | 25.08.2020 | 1.3E+08 | 1.1E+06 | 4.1E+03 | < LOQ | < LOQ | < LOQ | < LOQ | < LOQ | < LOQ | n.a. |
|  | 20.10.2020 | 1.5E+09 | 8.1E+05 | 3.5E+04 | < LOQ | < LOQ | < LOQ | < LOQ | < LOQ | < LOQ | < LOQ |
|  | 01.12.2020 | 8.3E+08 | 6.4E+05 | 7.2E+03 | < LOQ | < LOQ | 3.1E+03 | < LOQ | < LOQ | 2.6E+03 | < LOQ |

**Supplementary Table S5** Results of the Micronaut-S assay for 37 isolates from BL2 media. N.e. means that the resistance cannot be evaluated as another β-lactamase activity was already confirmed.

**Supplementary Table S6** Results of the Micronaut-S assay for 7 isolates from R2A media without β-lactams. n.e. means that the resistance cannot be evaluated as another β-lactamase activity was already confirmed.
